# Advancing maternal and newborn healthcare measurement: developing quality of care indices for postnatal and small and/or sick newborn care in low- and middle-income countries

**DOI:** 10.7189/jogh.15.04261

**Published:** 2025-11-21

**Authors:** Ashley Sheffel, Shannon King, Louise Tina Day, Tanya Marchant, Moise Muzigaba, Jennifer Requejo, Emily Carter, Melinda K Munos

**Affiliations:** 1 Department of International Health, Johns Hopkins University Bloomberg School of Public Health, Baltimore, Maryland, USA; 2 Department of Infectious Disease Epidemiology and International Health, London School of Hygiene & Tropical Medicine, London, UK; 3 Department of Maternal, Newborn, Child, and Adolescent Health and Ageing, World Health Organization, Geneva, Switzerland; 4 Global Financing Facility, World Bank, Washington, D.C., USA

## Abstract

**Background:**

High-quality healthcare for pregnant women and newborns, particularly postnatal care (PNC) and small and/or sick newborn care (SSNC), is essential to reducing maternal and newborn morbidity and mortality in low- and middle-income countries (LMICs). Poor quality of care (QoC) is a major contributor to preventable morbidity and mortality, emphasising the need for its improvement in health service delivery through systematic measurement and monitoring. Although indicators measuring QoC have been identified, there is a current gap in the availability of composite indicators that can summarise its complex, multidimensional nature. Here we present three systematically developed composite QoC indices for maternal PNC, newborn PNC, and SSNC, feasible to measure using existing data in LMICs.

**Methods:**

We developed a four-step process to define the indices. First, we identified interventions by reviewing global clinical guidelines and QoC frameworks. Second, we extracted discrete items recommended for delivery of each of the selected interventions from intervention-specific guidelines. Third, we mapped these items to health facility survey data to assess their alignment with standardised tools. Finally, we developed a quality readiness index (QRI) for each service area based on QoC frameworks, available data, and clinical guidelines.

**Results:**

The maternal PNC-QRI includes 12 interventions and contains 24 items, the newborn PNC-QRI includes three interventions and contains 16 items, and the SSNC-QRI includes eight interventions and contains 48 items. Data gaps across all three indices led us to exclude some evidence-based interventions and include a limited number of items. No data on provision/experience of care were available for maternal PNC, newborn PNC, or SSNC, so the indices reflect only facility readiness.

**Conclusions:**

The three QRIs provide composite measures for maternal and newborn PNC and SSNC readiness that could be adapted at the country level and operationalised using health facility assessment survey data, facilitating their use by decision-makers for planning and resource allocation. Revision of existing health facility assessments to address gaps in readiness and provision/experience of care measurement for PNC and SSNC would bolster efforts to monitor and improve care quality for mothers and newborns.

High-quality health services are crucial to achieving maternal, newborn and child health (MNCH) goals, including Sustainable Development Goal 3, which aims to ensure healthy lives and promote well-being for everyone at all ages [1,2]. Recognising the importance of these services, increasing emphasis is being placed on improving, measuring, and monitoring both access to and the quality of MNCH services. In this regard, a significant initiative launched in 2014 is the Every Newborn Action Plan (ENAP), which is a comprehensive, multi-partner effort calling on stakeholders to improve service access and quality of care (QoC) for all pregnant women and newborns. This initiative underscores the need for enhanced measurement, particularly concerning QoC, which is one of the strategic objectives [3]. However, a lack of standardised indicators for effectively monitoring maternal and newborn QoC means that service contact coverage indicators remain common for monitoring progress in service delivery.

While service contact coverage indicators such as postnatal care contacts for mothers and newborns within two days of delivery provide valuable information on access to health services, previous research has shown a contact-content coverage gap, where these indicators do not capture the specific interventions delivered or QoC provided during the service contact [4,5]. Monitoring efforts for maternal and newborn health have revealed a concerning trend: despite substantial improvements in service contact coverage, many countries are not achieving rapid reductions in maternal and newborn mortality [6]. This finding highlights that providing and monitoring high-quality maternal and newborn health services, including small and/or sick newborn care (SSNC), *i.e.* care for newborns who are small (weighting <2500 g at birth) or sick (have medical or surgical conditions during the neonatal period (days 0–28) among babies of all birthweights [7]), or routine postnatal care (PNC), *i.e.* care for mothers and newborns from immediately after birth through six weeks postpartum, [8] is critical to reducing maternal and neonatal morbidity and mortality in low- and middle-income countries (LMICs).

Measuring maternal and newborn QoC requires a clear definition thereof, along with standardised indicators and data sources for its operationalisation. The World Health Organization (WHO) has developed a definition for ‛quality of care’ – ‘the extent to which healthcare services provided to individuals and patient populations improve desired health outcomes. To achieve this, healthcare needs to be safe, effective, timely, efficient, equitable, and people-centred’ – and a framework for improving the QoC for mothers and newborns around the time of childbirth [9]. In addition, the WHO has published standards for improving quality of maternal and newborn care in health facilities, including for small and/or sick newborns, which contain quality standards, quality statements, and quality measures (350 quality measures for maternal and newborn health; 578 quality measures for small and/or sick newborn care) [7,10]. The maternal and newborn health QoC monitoring framework recognises stakeholders different measurement needs and proposes several measurement components, including a core set of indicators (a small set of prioritised input, process, outcome, and impact indicators to track and compare across and within regions and countries) and a quality improvement indicator catalogue (a menu of indicators to support quality improvement at facility and subnational levels) [11].

Although indicators for measuring QoC have been identified, there remains a gap in the availability of composite indicators that can summarise its complex and multidimensional nature. Composite indicators are formed when individual indicators are combined into a single index, which can be useful for assessing and monitoring overall health system progress and benchmarking within and across countries [12–14]. Those related to service quality are particularly useful for measuring and tracking effective coverage (EC) – the proportion of a population in need of a service that received the service with sufficient quality to achieve a positive health outcome – through the use of EC cascades. One common approach to estimating EC is to link composite indicators for service readiness and process quality to measures of service contact coverage [4,15–18]. Utilising existing data generated from commonly implemented health facility assessments (HFAs) in LMICs which are designed to assess the quality of services, such as the service provision assessment (SPA), service availability and readiness assessment (SARA), and harmonised health facility assessment (HHFA), provides an efficient, sustainable way to measure both composite quality indicators and support effective coverage measurement [19–22]. Given the need for composite indicators that can be measured with data currently available in LMICs, this study aimed to systematically develop QoC indices for maternal PNC, newborn PNC, and SSNC using existing HFA data.

## METHODS

We used a four-step process, similar to an approach previously taken for developing QoC indices for maternal nutrition, to define QoC indices for PNC for women, PNC for newborns, and SSNC in LMICs [23]. This was a theoretically driven approach to index development and not a data-driven approach utilising data from specific countries, with the process involving:

1. Intervention selection: we reviewed global clinical guidelines and QoC frameworks to select recommended interventions.
2. Guideline review and item identification: we reviewed intervention-specific clinical and service implementation guidelines to identify discrete elements or ‘items’ recommended for delivery of each of the selected interventions using the WHO maternal and newborn health QoC framework as an organising framework.
3. Data mapping: we matched the identified discrete items to available health facility survey data, assessing the degree of alignment with standardised health facility assessments.
4. Final index development: we developed final QoC indices for each service area informed by QoC frameworks, clinical guidelines, and data availability.

The SSNC index development process was funded through a separate mechanism from the PNC work, with more limited objectives, focussing solely on readiness, and not provision/experience of care. Therefore, for SSNC, we implemented the above process solely for readiness, whereas for maternal and newborn PNC, we extended the approach to include the provision/experience of care. We also note that large HFA programmes, including the SPA, SARA, and HHFA, do not currently collect provision or experience of care data for SSNC.

### Intervention selection

We identified maternal and newborn PNC interventions through a review of the 2022 WHO recommendations on maternal and newborn care for a positive postnatal experience [8]. We identified interventions for SSNC through a review of WHO guidelines and previous studies assessing facility readiness for SSNC [7,24–26].

### Guideline review and item identification

We included PNC interventions in guideline review and item identification if the WHO recommended the intervention either for all or for specific contexts, and if the intervention was a clinical intervention. We excluded PNC interventions if the intervention was not recommended by WHO or was a best practice rather than a clinical intervention. We included SSNC interventions in guideline review and item identification if they were routine and essential newborn care or special newborn care clinical interventions. Since we aimed to use existing data, we excluded SSNC interventions if they were recommended at the intensive care level or transition to intensive newborn care, as these are highly specialised services, which are not collected in the SPA/SARA/HHFA.

Through the guideline extraction step, we aimed to identify discrete elements or ‘items’ recommended for delivery of each of the selected interventions. For each intervention that met the inclusion criteria, we first reviewed WHO facility-level service delivery guidelines; where those were lacking, we hand-searched the references from the key documents used to identify interventions in step 1 and identified and reviewed other available guidance and protocols (*e.g. Médecins Sans Frontières*, the American Academy of Pediatrics, country-specific guidelines), and/or published peer-reviewed literature (Table S2 in the **Online Supplementary Document**). We organised the guideline extraction by the quality domains proposed by the WHO QoC framework for maternal and newborn health, including provision of care, experience of care, and service readiness (**Box 1**) [10,27]. We further categorised provision of care into the sub-domains of assessment, intervention, and documentation and referral, and service readiness into the sub-domains of basic amenities, equipment and supplies, medicines and commodities, diagnostics, guidelines and staff training, and, for SSNC only, routine service. Two researchers at Johns Hopkins University (SK and AS) conducted the guideline extraction process.

Box 1 **Key definitions of quality dimensions**

- Provision of care refers to the quality of delivery of interventions by providers to clients (*i.e.* the content of care), which includes following evidence-based practices for routine care and management of complications.

- Experience of care refers to the client's experience, including effective communication by the care provider about the services provided, client expectations, and client rights; care provided with respect and preservation of dignity; and client access to emotional and social support of their choice.

**-** Service readiness refers to the capability of health facilities to provide a service of minimum acceptable standards and is measured by the availability of both physical resources and human resources.

**-** Routine service includes whether the facility reports delivering key interventions. We have included routine service as a sub-domain for SSNC to make up for the lack of data in other readiness sub-domains for SSNC interventions. If more readiness data were available, the routine service sub-domain could be excluded from the index, as it reflects historical service delivery rather than service readiness.

### Data mapping

The SPA, SARA, and HHFA are three of the most widely implemented HFAs in LMICs and provide nationally representative data on health service delivery, including service readiness across the continuum of care and provision/experience of care for select services [28–30]. We selected these surveys because developing QoC indices using the data available from these surveys provides a means to operationalise them using existing data in LMICs. We matched each item identified during the guideline extraction process with available items from the SPA and SARA standard questionnaires. Both SPA and SARA were updated in 2022, with the SARA replaced by the HHFA [19–22,31]. As such, we mapped to the older questionnaires, which correspond to existing country data, as well as the newer questionnaires, which represent data that will be available from future country surveys. We classified the level of agreement between the item in the guideline and the item in the HFA questionnaire as an exact match, high/low partial match, or nonmatch (**Box 2**). All items that were an exact match, high partial match, or low partial match were eligible for inclusion in the QoC indices.

Box 2 **Definition of exact, partial, and nonmatch**

- Exact matches were items from the guidance documents for which an exact item was available within at least one of the HFA questionnaires.

- Partial matches were items for which a partially matching item was available within at least one of the HFA questionnaires. Partial matches were separated based on the specificity of the HFA item compared to the guidance document. For example:

• For specific intervention guidelines (*e.g.* newborn assessment) from the guidance document, a high partial match in the HFA would be broad service areas guidelines that explicitly include that intervention (*e.g.* guidelines for IMPAC).

• For the item ‛staff trained in administering paracetamol within the context of perineal pain relief’, the HFA indicator of staff trained in a broad service area package (*e.g.* staff trained in IMPAC) was considered a low partial match because it was not clear whether the specific intervention was included in training.

- Nonmatches were items for which there was no appropriate match within any HFA questionnaires.

### Final index development

We excluded PNC and SSNC interventions in final index development if data were insufficient (*i.e.* no matching items available or the key equipment, commodity, diagnostic or human resource item required to deliver the intervention was not available) or if the intervention was combined with another intervention due to overlap of the content of care. We aimed to develop QoC indices that reflected recommended interventions based on the latest WHO guidelines for the three subpopulations – women who recently delivered, newborns, and SSNs – and the items required to deliver those interventions. As such, the QoC indices include all interventions that met the inclusion criteria and all items within those interventions that were an exact match, a high partial match, or a low partial match. We reviewed exact and partial match items across interventions and identified overlapping items. We combined interventions if all matching items from one intervention were also included in another. Furthermore, we examined the balance of items across interventions and combined some items into a single indicator (*e.g.* immunisation supplies, available HIV guidelines and staff training, training in integrated management of pregnancy and childbirth (IMPAC) or newborn care) to prevent any single intervention from dominating the indices (Table S3 and Table S5 in the **Online Supplementary Document**).

We assessed possible methods for combining the index items, including a simple average, weighted averages (weighting by either intervention or QoC sub-domain), and data-driven approaches, such as principal component analysis, latent class analysis, and item response theory. We ultimately excluded data-driven approaches, as they often resulted in indices that did not reflect conceptual frameworks and clinical knowledge of QoC [32–34]. We considered the distribution of items within QoC sub-domains and within interventions to decide on a simple or weighted average approach to calculating the index scores for each service area. If the number of items was similar in each sub-domain, we opted for a simple average. Otherwise, a weighted average was used with the option to utilise a sub-domain weighted approach or an intervention-weighted approach. Finally, where we used an intervention-weighted approach, we reviewed items across interventions to identify general items that were required for multiple interventions. We moved these items to a separate general intervention area to prevent double-counting of items across interventions within the index.

## RESULTS

### Identification of interventions, guideline review and item identification

**Figure 1:**
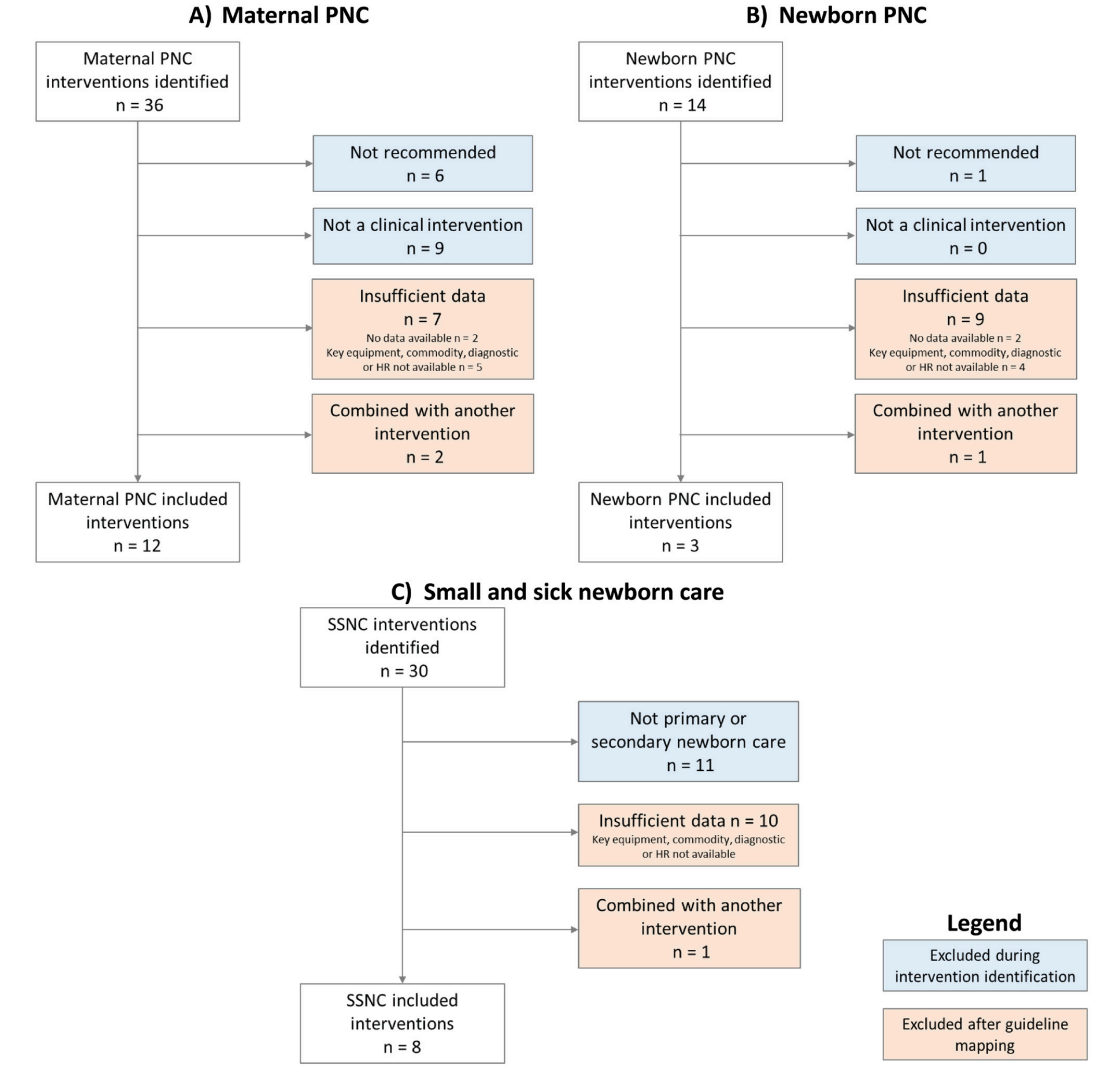
Intervention selection. **Panel A.** Maternal postnatal care. **Panel B.** Newborn postnatal care. **Panel C.** Small and/or sick newborn care. HR – human resource. PNC – postnatal care. SSNC – small and/or sick newborn care.

For maternal PNC, we identified 36 interventions with 21 meeting the criteria for guideline review and item identification. We excluded WHO non-recommended interventions (n = 6) and non-clinical interventions (n = 9). For newborn PNC, we identified 14 interventions, with 13 meeting the inclusion criteria for guideline review and item identification, and excluded one non-recommended intervention. For SSNC, we identified 30 interventions, with 19 meeting the inclusion criteria for guideline review and item identification, and excluded those for intensive-care (n = 11) (**Figure 1**, Table S1 in the **Online Supplementary Document**).

We conducted guideline extraction for the 21 maternal PNC, 13 newborn PNC, and 19 SSNC interventions. There was variability in the total number of items required for each intervention as well as the sub-domains across which those items were located (Table S2 in the **Online Supplementary Document**).

### Data mapping

#### Overview of mapping and quality of alignments

The review of the SPA and SARA showed that provision and experience of care data collected through direct observation and client exit interviews were limited to a few select services (antenatal care, family planning, curative care for sick children). While the updated 2023 SPA contains an exit interview for PNC clients, it only covers topics related to counselling and experience of care, which is insufficient to develop a full provision/experience of care index. While there is evidence of the validity of maternal report of certain PNC interventions through exit interviews, those interventions were largely not included in the SPA exit interview [35,36]. The lack of provision and experience of care data resulted in no matching items for these quality domains (Table S6–8 in the **Online Supplementary Document**).

There were 89 full or partial matches and 40 nonmatches for maternal PNC across the 21 included maternal interventions from the WHO PNC guidelines [8], 44 full or partial matches and 48 nonmatches for newborn PNC across the 13 included newborn interventions from the WHO PNC guidelines [8], and 167 full or partial matches and 130 nonmatches for SSNC across the 19 interventions included from the review of WHO guidelines and previous studies assessing facility readiness for SSNC [7,24–26].

Many of the maternal and newborn PNC exact matches were service readiness items required to provide health services in general, but were not specific to PNC (*e.g*. infection prevention and control items, power, vaccines, diagnostics for tuberculosis and HIV, thermometer, stethoscope). Partial and nonmatches reflected common limitations in the SPA and SARA. For example, we found a lack of items on specific PNC training topics and a lack of specificity about guideline content for PNC services. No dosage information was captured in SPA and SARA for medicines/commodities, although this is required to determine readiness for interventions by age group (*e.g.* neonatal and maternal vitamin A supplementation and iron supplementation). In addition, the SPA and SARA did not include some PNC-specific commodities (*e.g.* vitamin D, massage oil). PNC-specific equipment and supplies, such as materials required to conduct universal screenings (*e.g.* for hearing, eye abnormalities, neonatal hyperbilirubinemia) and education materials were also not captured in the SPA and SARA questionnaires.

For SSNC, exact matches included service readiness items required to provide health services generally (*e.g.* power, emergency transportation, haemoglobin testing, full blood count, antiretrovirals, vaccines) as well as some SSNC-specific items (*e.g.* neonatal bag and mask device, weighing scale, first-line antibiotics). Partial and nonmatches reflected a lack of special newborn care clinical interventions for SSNC in the SPA and SARA questionnaires. In addition, the SPA and SARA did not collect some SSNC-specific commodities at all (*e.g.* vitamin K, oral sucrose, phenobarbital, methylxanthines). SSNC-specific equipment and supplies were also limited in scope in the SPA and SARA questionnaires. Moreover, similar to PNC, there was a lack of items on specific SSNC training topics and insufficient specificity in guideline content for SSNC services.

#### Intervention inclusion and exclusion based on data availability

For maternal PNC, we retained 12 interventions for inclusion in the maternal PNC quality readiness index (QRI) and excluded seven interventions due to having insufficient data (**Figure 1**, Table S6 in the **Online Supplementary Document**). Two interventions had no matching items in the HFAs, and five interventions were missing the key equipment, commodity, diagnostic or human resources required to deliver the intervention. Those excluded based on data availability were often counselling-based or required only trained staff. Furthermore, we excluded two interventions (non-pharmacological interventions to prevent breast engorgement and postpartum mastitis) as they were combined with another intervention (counselling and support for exclusive breastfeeding) due to overlap in the content of care and required readiness items with available data.

For newborn PNC, we retained three interventions for inclusion in the newborn PNC-QRI (Table S7 in the **Online Supplementary Document**) and excluded 10 interventions due to having insufficient data (**Figure 1**). Four interventions had no matching items in the HFAs, and six interventions were missing the key equipment, commodity, diagnostic or human resources required to deliver the intervention. Interventions we excluded based on data availability were often universal screenings that required specific equipment or interventions requiring specific commodities that were not available in the HFAs.

For SSNC, we retained eight interventions in the SSNC-QRI (Table S8 in the **Online Supplementary Document**). We found an overlap between newborn PNC and SSNC interventions. This is likely because many small and/or sick newborns also need essential PNC services (*e.g.* prevention of mother-to-child transmission (PMTCT), immediate newborn care, early initiation and breastfeeding support, pre-discharge advice on mother and baby care and follow up, detection and management of jaundice). We excluded 11 interventions due to insufficient data (**Figure 1**), which were often services expected to be available at hospitals with specialised newborn care units. All these interventions were missing the key equipment, commodity, diagnostic or human resources required to deliver the intervention.

#### Mapping and quality of alignment with the new SPA and HHFA

Both SPA and SARA surveys had a few notable limitations for measuring the quality of PNC and SSNC services. Neither facility survey included a specific service area module for collecting data on PNC or SSNC. Instead, these instruments relied on modules covering other service areas where PNC and SSNC services may be delivered (*e.g.* childbirth, child well-visits, HIV/AIDS) to gather information about readiness to deliver PNC and SSNC. In addition, the SPA and SARA do not collect any provision and experience of care items for PNC and SSNC. However, both surveys were revised in 2022, introducing a new SPA questionnaire and the HHFA as the successor to the SARA [21,31]. We repeated the mapping exercise using the new versions of the SPA and HHFA questionnaires (Table S6–8 in the **Online Supplementary Document**). We found that, in general, the HHFA expanded to include a specific section for PNC and SSNC, along with additional medicines, equipment, and supplies for interventions such as thermal care, and inclusion of more specific PNC guidelines and staff training. In comparison, the SPA largely contracted towards a reduced number of items to serve as a more streamlined tool with no specific PNC or SSNC sections. These changes mean that, for the maternal PNC-QRI, the HHFA would include one additional context-specific intervention (preventive schistosomiasis treatment), while the SPA would exclude four interventions, due to items being dropped from the new survey version. For the newborn PNC-QRI, the only change if using the HHFA survey would be the ability to include timing of first bath to prevent hypothermia and its sequelae. Mapping to the new SPA and HHFA questionnaires resulted in more changes for the SSNC-QRI. The HHFA would allow for the inclusion of four additional interventions, while the SPA would do so for three additional interventions, two of which are the same as the HHFA. However, the new SPA would also result in the exclusion of two interventions, PMTCT and kangaroo mother care.

### Final index development

No provision/experience of care data was available for PNC or SSNC; thus, the indices reflect facility readiness only. After we excluded interventions with insufficient data, limited data was available to generate readiness indices for maternal PNC, newborn PNC, and SSNC. We therefore did not conduct an expert survey to prioritise interventions or items within interventions, as has been done for other service areas [23,33,37]. Instead, we included all available items based on the older SPA and SARA survey mapping for each included intervention in the indices. We also examined the balance of items across interventions and combined some items into a single indicator to ensure the indices were not dominated by any one intervention (*e.g.* immunisation supplies, available HIV guidelines and staff training, training in IMPAC or newborn care (Tables S3–5 in the **Online Supplementary Document**). We retained and denoted context-specific interventions and associated items in the QRIs to allow operationalisation based on country policy. For the maternal and newborn PNC-QRIs, there was an unequal distribution of items across sub-domains, and similar individual items across interventions; thus, we opted for a sub-domain-weighted approach with the sub-domains corresponding to basic amenities, equipment and supplies, medicines and commodities, diagnostics, and guidelines and staff training. In contrast, for the SSNC-QRI, items were unevenly distributed across sub-domains and interventions; thus, we opted for the intervention-weighted approach. It is also important to note that interventions required for small newborns may differ from those needed for sick newborns. While we included all interventions together in this index, examining the quality of individual interventions may be important when assessing readiness for small newborns separately from sick newborns.

The maternal PNC-QRI includes 24 items, of which eight items are context-specific, across five sub-domains: 11 equipment and supplies (of which five are infection prevention and control-related items), five medicines and commodities, two diagnostics, one basic amenity, and five guidelines and staff training (**Table 1**). The newborn PNC-QRI includes 16 items (of which one is context-specific) across three sub-domains, as follows: eleven on equipment and supplies (of which five are infection prevention and control-related items), two on medicines and commodities, and three on guidelines and staff training (**Table 2**). The SSNC-QRI includes 48 items, of which nine are context-specific, across eight interventions plus an intervention for general/cross-cutting readiness items (**Table 3**) The interventions with the most items are immediate newborn care and routine care (11 items), PMTCT (eight items), and general readiness items (12 items), while the interventions with the fewest items are early initiation and breastfeeding support (two items), comfort and pain management (two items), and detection and management of hypoglycaemia (two items).

**Table 1:** Proposed items in the maternal PNC-QRI by readiness sub-domains

Equipment and supplies, n = 11	Medicine and commodities, n = 5	Diagnostics, n = 2	Basic amenities, n = 1	Guidelines and staff training, n = 5
Blood pressure apparatus	Supplement containing iron*	HIV diagnostic capacity*	Room is private room with auditory and visual privacy	Guidelines containing information on pregnancy (*e.g.* IMPAC)
Stethoscope	Albendazole or mebendazole*	TB diagnostic capacity*		Training in IMPAC
Thermometer	Pre-exposure prophylaxis*			Training in early and exclusive breastfeeding
Sputum collection container*	Mix of family planning methods			Available HIV guidelines and staff training*
Single-use standard disposable syringes with needles or auto-disable syringes with needles	Paracetamol			Available TB guidelines and staff training*
Environmental disinfectant				
Gloves				
Non-sharps waste (pedal bin receptable with lid and plastic liner)				
Sharps container				
Soap and water for handwashing/alcohol based handrub				
Examination light				

IMPAC – integrated management of pregnancy and childbirth, TB – tuberculosis

*Context-specific inclusion based on country policy.

**Table 2:** Proposed items in the newborn PNC-QRI by readiness sub-domains

Equipment and supplies, n = 11	Medicine and commodities, n = 2	Diagnostics, n = 0	Basic amenities, n = 0	Guidelines and staff training, n = 3
Stethoscope	Chlorhexidine solution*			IMPAC or ENC guidelines
Thermometer	Vaccines (BCG, Hep B, OPV0)			Child vaccination guidelines
Single-use standard disposable syringes with needles or auto-disable syringes with needles				Training in IMPAC or newborn care
Vaccine documentation (blank/unused individual child vaccination cards or booklets, and immunisation tally sheet)				
Refrigerator with temperature monitoring device and power; or vaccine carrier with ice packs				
Gloves				
Environmental disinfectant				
Non-sharps waste (pedal bin receptable with lid and plastic liner)				
Room with auditory and visual privacy				
Soap and water for handwashing/alcohol based handrub				
Sharps container				

ENC – essential newborn care, IMPAC – integrated management of pregnancy and childbirth

*Context-specific inclusion based on country policy.

**Table 3:** Proposed items in the SSNC-QRI by intervention and readiness sub-domains

	Immediate newborn care and routine care, n = 11	Early initiation and support for breastfeeding, n = 2	Neonatal resuscitation, n = 4	PMTCT of HIV, n = 8	KMC, n = 3	Detection and management of neonatal infection, n = 4	Comfort and pain management, n = 2	Detection and management of hypoglycaemia, n = 2	General readiness items, n = 12
**Basic amenities**				PMTCT room is private room with auditory and visual privacy*	Separate room or space for KMC				
**Equipment and supplies**	Linen for drying baby; cord cutting supplies, thermometer for low-body temperature; radiant heater/warmth source		Airway suction apparatus; infant resuscitation bag/mask						Thermometer; infant scale, stethoscope; medication delivery mechanism; pulse oximeter; oxygen supply, non-sharps waste container; environmental disinfectant; clean/sterile gloves; sharps container; handwashing supplies
									
**Medicines and commodities**	Vitamin K			Antiretrovirals for newborns*		Antibiotic treatment for neonatal infection	Paracetamol	Glucose injectable solution	
	Antibiotic eye ointment			Antiretrovirals for mothers*			Morphine		
	Chlorhexidine solution*			Cotrimoxazole*					
	Immunization supplies								
**Diagnostics**				HIV diagnostic capacity*		Full blood count; chest x-ray		Blood glucose testing capacity	
**Guidelines and staff training**	Staff trained in clean cord cutting and appropriate cord care	Staff trained in early and exclusive breastfeeding	Staff trained in neonatal resuscitation using bag and mask	PMTCT and IYCF guidelines*	Staff trained in KMC for low-birth-weight babies	Staff trained in newborn infection management			Guidelines containing information on newborn care (*e.g.* ENC, IMPAC, BEmOC, CEmOC)
	Staff trained in thermal care			Staff trained PMTCT*					
				Staff trained in newborn nutrition counseling of mother with HIV or IYCF*					
**Routine service**	Routine, complete examination of newborn before discharge	Initiation of breastfeeding within the first hour	Facility past three months provided neonatal resuscitation		Facility practices KMC				

ENC – essential newborn care, KMC – kangaroo mother care, IMPAC – integrated management of pregnancy and childbirth, IYCF – infant and young child feeding, PMTCT – prevention of mother-to-child transmission

*Indicates context-specific inclusion based on country policy

## DISCUSSION

We describe the development of three QRIs that can provide standardised measures for maternal PNC, newborn PNC, and SSNC service readiness and can be adapted at the country level and operationalised using existing HFA data, facilitating their use by decision-makers for planning and resource allocation. A lack of data availability in HFAs meant that we could not develop summary indices of service provision or experience of care. We also found substantial gaps in the readiness data, even after reviewing the recently revised SPA and HHFA.

There have been a few attempts to systematically develop indices of QoC for maternal and newborn health services that have carefully described their methods and assumptions, which are focussed on family planning, antenatal care, nutrition, and childbirth care [23,32–34]. These approaches are similar to the one described here for developing summary QRIs for maternal PNC, newborn PNC, and SSNC in that they utilise a systematic approach to index development, are rooted in the latest guidelines and guidance, and explore multiple approaches to item selection and index aggregation methods. Much more commonly, research studies exploring access to quality services and/or associations between quality and other outcomes (*e.g.* many of the studies included in the Do *et al.* and Sheffel *et al.* reviews [28,38]) use summary measures of service quality, but the methods for their development are secondary to the primary research question. As a result, there is substantial variability in the methods employed, including item inclusion and aggregation methods, largely due to the lack of guidance on best practices. Thus, it is difficult to synthesise learnings across these studies, as they may not be comparable due to inconsistencies in measurement approaches. Our work on developing summary QRIs for maternal PNC, newborn PNC, and SSNC, utilising a systematic approach to identifying interventions and items and guided by up-to-date clinical guidelines, contributes to the growing evidence around generating summary measures of service quality.

The data mapping process, which assessed data gaps across two versions of two different HFAs (the SARA/HHFA and the SPA), highlighted limitations of existing HFAs to characterise service readiness for PNC and SSNC. This finding echoes those of other studies, which have noted the need to align existing measurement tools with global standards in order to fill gaps in QoC measurement [28,29]. We found that publicly available HFAs have not historically included a PNC or SSNC module; hence, they have had very limited readiness data for the target groups. However, the new HHFA does have PNC and SSNC modules, and additional readiness items for these areas have been incorporated, which will be beneficial for assessing service readiness moving forward. We also found that some recommended interventions were completely omitted from HFAs. Our SSNC-QRI could not include any intensive level interventions, as HFAs have primarily been designed to collect information at primary/secondary-level healthcare facilities. The PNC interventions we excluded due to data insufficiency were often counselling-based and required only trained staff, or universal screening (*e.g.* hearing or eye abnormality) that required specific equipment. We were also limited to developing only service readiness indices, as there is no direct observation of PNC or SSNC in the SPA/SARA/HHFA to enable measurement of process quality. Finally, many of the exact matches found in the existing HFAs were service readiness items required to provide health services generally and were not specific to PNC and SSNC. This finding may have implications for the ability of these indices to differentiate facilities with high and low readiness for PNC and SSNC. Research has shown that indices generated with relatively few items are prone to ties across facilities and ceiling effects, particularly when many of the items are almost universally available at health facilities [34]. In the absence of a validated set of tracer indicators, efforts to strengthen the comprehensive measurement of maternal PNC, newborn PNC, and SSNC readiness are warranted, with a focus on including items in HFAs based on clinical considerations and the ability to discriminate between levels of service readiness.

Both the HHFA and SPA are revised at regular intervals, with many factors considered when determining which items to include in an HFA inventory. For future iterations, global survey programmes could consider incorporating additional readiness items specifically tailored to PNC and SSNC to better capture these critical aspects of care. We recommend conducting an expert survey to prioritise key items for inclusion, ensuring that selected indicators reflect both clinical relevance and the ability to meaningfully differentiate service readiness levels across facilities. However, collecting comprehensive provision of care data presents significant challenges. For instance, direct observation methods, such as observing neonatal resuscitation or other SSNC interventions, require the presence of cases, which can be rare and logistically complex to capture systematically. Similarly, vignettes may be a feasible option for assessing specific skills, such as neonatal resuscitation, but scaling this approach across the range of PNC and SSNC interventions would be difficult. Record reviews, while potentially valuable, can be hindered in settings without standardised documentation practices. Individual studies and evaluations have demonstrated success in using these methods to collect provision of care data for specific interventions [36,39–47], suggesting that targeted studies rather than broad global surveys may be the most practical approach for obtaining comprehensive data on PNC and SSNC provision of care. While global surveys like the HHFA and SPA are invaluable for capturing broad trends in readiness and quality, expecting them to comprehensively collect provision or experience of care data, particularly for specialised areas such as SSNC, may not be feasible. Instead, supplementing these surveys with focussed evaluations or studies could offer a more practical way forward.

The main advantage of a summary measure is to allow monitoring of progress and comparisons of the levels and trends in service readiness for maternal and newborn care at national level [48]. Although composite measures like QRIs do not provide information on which specific items are lagging behind, this information can readily be obtained by policymakers and stakeholders at a national and sub-national level if needed to inform targeted interventions and resource allocation. Summary measures of service readiness may also be useful for conducting effective coverage analyses that examine readiness (*i.e.* input-adjusted coverage) as a key step in the effective coverage cascade [49]. However, summary indices may be less useful at the facility level, where more granular information may be required to identify specific problem areas for quality improvement. Our proposed maternal PNC, newborn PNC, and SSNC QRIs will require country adaptation, especially for context-specific interventions, which may make cross-country comparisons more difficult at regional or global levels. This adaptation is crucial to ensure that the indices accurately reflect a country's policies, health system practices, and implementation realities. Moreover, this need for adaptation introduces a tension between customisation and comparability. The more an index is tailored to a specific context, the less comparable it becomes across countries. For instance, some countries may prioritise the use of chlorhexidine for newborn umbilical cord care as part of their essential interventions, while others may not, due to differences in epidemiological contexts or policy frameworks. If the goal is cross-country comparability, some level of standardisation will be necessary, which may require limiting the extent of country-specific adaptation. Balancing this trade-off is essential to ensure that the indices are both actionable at the country level and meaningful for regional or global comparisons.

The study has several limitations that should be acknowledged. First, the reliance on existing data sources limited the scope of the indices, as some interventions and domains of quality could not be adequately captured. One key challenge with the SSNC-QRI is the exclusion of intensive/transition interventions (*e.g.* continuous positive airway pressure) from the readiness index. These interventions are linked to ENAP coverage measures; however, we could not include these interventions in our QRI due to data availability gaps. Although most small and/or sick newborns do not require intensive care, expansion of HFAs to capture these interventions may help fill this data gap. It may also be difficult to incorporate these intensive-level interventions into a QRI, as it will require facilities at distinct levels to have different indices to account for differentials in expected service delivery. Hence, using existing HFA data to improve measurement for intervention readiness that will benefit the greatest number of SSNs can be prioritised now, and intensive-level interventions added into further versions of these HFA tools. The current data availability gaps for provision and experience of care also limited our ability to assess the technical delivery of PNC and SSNC interventions and the experiences of mothers and newborns. As a result, the indices we developed represent a set of items that can be measured with existing HFA data to facilitate country use of these measures, given current data constraints. However, they are not representative of readiness to deliver complete maternal/ newborn PNC or SSNC services. Second, we did not conduct an expert survey to prioritise readiness items for inclusion in the indices, which would strengthen the face validity of the indices. There were few items available in existing HFAs; thus, there was no need to prioritise the available items. However, we utilised a guideline-driven approach to item selection, prioritising recently published service guidelines such as the WHO PNC guidelines [8], standards for improving the quality of care for small and sick newborns in health facilities [7], and the WHO framework for the provision of quality maternal and newborn care [9], which were developed through an extensive literature review and expert consultations. Third, while the indices we designed measure what should be happening in health service delivery, their generalisability may vary across countries due to differences in health systems and implementation of interventions, which can affect their applicability. While we have proposed a single index based on guidelines and data availability, adaptation and validation of the indices at the country level is necessary to ensure they are suitable for the setting in which they are used. Fourth, we mapped data elements to existing HFAs and did not explore alternative data sources, such as routine health management information system (HMIS) data, which have the potential to contribute to maternal and newborn QoC measurement. However, existing reviews of HMIS in LMICs have highlighted significant limitations, noting that indicators for measuring and improving quality of care are not consistently available in HMIS data across countries [50–52]. Finally, we were not able to assess construct validity by, for example, examining the association with provision of care or health outcomes.

Our work has highlighted several critical areas for future research. Future efforts to develop an ideal summary measure of service readiness and provision/experience of care for maternal PNC, newborn PNC, and SSNC without consideration of data availability would be helpful to clearly identify data gaps. In addition, identifying a smaller set of salient interventions that are strongly associated with leading causes of death or complications or a small set of items within interventions that are strongly associated with service quality or health outcomes would be helpful to reduce the overall number of items in the index, and thus the data collection burden, and could potentially inform the weighting of items in the index. A more focussed set of interventions and items may facilitate measurement of maternal PNC, newborn PNC, and SSNC service quality in HFAs, which must balance comprehensiveness with implementation feasibility. Finally, exploration of alternative data sources such as routine data to generate summary measures of maternal PNC, newborn PNC, and SSNC service readiness and provision/experience of care would be useful in supporting more regular measurement at the country level.

## CONCLUSIONS

Use of improved data on service readiness and service provision is needed to enhance quality of maternal and newborn care. Our summary indices provide a valuable step towards measuring and monitoring service readiness for maternal PNC, newborn PNC, and SSNC in LMICs. The utilisation of existing data sources and a systematic approach to index development enhance the feasibility and applicability of these measures. The indices can inform policy and decision-making processes, allowing for targeted interventions and resource allocation to improve the quality of care received by mothers and newborns. Future research is needed to expand the scope of the indices by incorporating provision of care and experience of care domains. In addition, assessing the proposed indices for validity and reliability would strengthen their effectiveness in capturing the QoC provided to mothers and newborns. If the gaps in readiness and provision of care measurement are addressed, PNC and SSNC indices have the potential to drive improvements in the delivery of high-quality health services to women and newborns, ultimately contributing to the reduction of maternal and newborn mortality in LMICs.

## Additional material


Online Supplementary Document

## References

[1] United Nations. Goal 3: Ensure healthy lives and promote well-being for all at all ages – United Nations Sustainable Development. 2022. Available: https://sdgs.un.org/goals/goal3. Accessed: 28 June 2022.

[2] Mason E McDougall L Lawn JE Gupta A Claeson M Pillay Y From evidence to action to deliver a healthy start for the next generation. Lancet. 2014;384:455–67. 10.1016/S0140-6736(14)60750-9 24853599

[3] World Health Organization. Every newborn: an action plan to end preventable deaths. Geneva, Switzerland: World Health Organization; 2014. Available: https://www.who.int/initiatives/every-newborn-action-plan. Accessed: 16 October 2025.

[4] Amouzou A Leslie HH Ram M Fox M Jiwani SS Requejo J Advances in the measurement of coverage for RMNCH and nutrition: from contact to effective coverage. BMJ Glob Health. 2019;4:e001297. 10.1136/bmjgh-2018-001297 31297252 PMC6590972

[5] Kruk ME Larson E Twum-Danso NAY Time for a quality revolution in global health. Lancet Glob Health. 2016;4:e594–6. 10.1016/S2214-109X(16)30131-0 27539798

[6] Souza JP Gulmezoglu AM Vogel J Carroli G Lumbiganon P Qureshi Z Moving beyond essential interventions for reduction of maternal mortality (the WHO Multicountry Survey on Maternal and Newborn Health): a cross-sectional study. Lancet. 2013;381:1747–55. 10.1016/S0140-6736(13)60686-8 23683641

[7] World Health Organization. Standards for improving the quality of care for small and sick newborns in health facilities. Geneva, Switzerland: World Health Organization; 2020. Available: https://www.who.int/publications/i/item/9789240010765. Accessed: 16 October 2025.

[8] World Health Organization. WHO recommendations on maternal and newborn care for a positive postnatal experience. Geneva, Switzerland: World Health Organization; 2022. Available: https://www.who.int/publications/i/item/9789240045989. Accessed: 16 October 2025. 35467813

[9] Tunçalp Ӧ Were WM MacLennan C Oladapo OT Gulmezoglu AM Bahl R Quality of care for pregnant women and newborns-the WHO vision. BJOG. 2015;122:1045–9. 10.1111/1471-0528.13451 25929823 PMC5029576

[10] World Health Organization. Standards for improving quality of maternal and newborn care in health facilities. Geneva, Switzerland: World Health Organization; 2016. Available: https://www.who.int/publications/i/item/9789241511216. Accessed: 16 October 2025.

[11] World Health Organization. Quality of Care for Maternal and Newborn Health: A Monitoring Framework for Network Countries. Geneva, Switzerland: World Health Organization; 2019. Available: https://www.who.int/publications/m/item/quality-of-care-for-maternal-and-newborn–a-monitoring-framework-for-network-countries. Accessed: 16 October 2025.

[12] Nardo M, Saisana M, Saltelli A, Tarantola S, Hoffmann A, Giovannini E. Handbook on constructing composite indicators: methodology and user guide. Paris, France: OECD publishing; 2008. Available: https://www.oecd.org/content/dam/oecd/en/publications/reports/2008/08/handbook-on-constructing-composite-indicators-methodology-and-user-guide_g1gh9301/9789264043466-en.pdf. Accessed: 22 October 2025.

[13] Wilhelm D Lohmann J De Allegri M Chinkhumba J Muula AS Brenner S Quality of maternal obstetric and neonatal care in low-income countries: development of a composite index. BMC Med Res Methodol. 2019;19:154. 10.1186/s12874-019-0790-0 31315575 PMC6637560

[14] Profit J Typpo KV Hysong SJ Woodard LD Kallen MA Petersen LA Improving benchmarking by using an explicit framework for the development of composite indicators: an example using pediatric quality of care. Implement Sci. 2010;5:13. 10.1186/1748-5908-5-13 20181129 PMC2831823

[15] Marsh AD Muzigaba M Diaz T Requejo J Jackson D Chou D Effective coverage measurement in maternal, newborn, child, and adolescent health and nutrition: progress, future prospects, and implications for quality health systems. Lancet Glob Health. 2020;8:e730–6. 10.1016/S2214-109X(20)30104-2 32353320 PMC7196884

[16] Ng M Fullman N Dieleman JL Flaxman AD Murray CJ Lim SS Effective coverage: a metric for monitoring Universal Health Coverage. PLoS Med. 2014;11:e1001730. 10.1371/journal.pmed.1001730 25243780 PMC4171091

[17] Jannati A Sadeghi V Imani A Saadati M Effective coverage as a new approach to health system performance assessment: a scoping review. BMC Health Serv Res. 2018;18:886. 10.1186/s12913-018-3692-7 30470214 PMC6251131

[18] Kruk ME Gage AD Arsenault C Jordan K Leslie HH Roder-DeWan S High-quality health systems in the Sustainable Development Goals era: time for a revolution. Lancet Glob Health. 2018;6:e1196–252. 10.1016/S2214-109X(18)30386-3 30196093 PMC7734391

[19] Demographic and Health Surveys. SPA Overview. 2020. Available: http://dhsprogram.com/What-We-Do/Survey-Types/SPA.cfm. Accessed: 16 November 2020.

[20] O’Neill K Takane M Sheffel A Abou-Zahr C Boerma T Monitoring service delivery for universal health coverage: the Service Availability and Readiness Assessment. Bull World Health Organ. 2013;91:923–31. 10.2471/BLT.12.116798 24347731 PMC3845262

[21] World Health Organization. Harmonized Health Facility Assessment (HHFA): Introduction. 2021. Available: https://www.who.int/data/data-collection-tools/harmonized-health-facility-assessment/introduction. Accessed: 1 June 2023.

[22] World Health Organization. Service availability and readiness assessment (SARA). Available: https://www.who.int/data/data-collection-tools/service-availability-and-readiness-assessment-(sara)?ua=1. Accessed: 1 July 2021.

[23] King SE Sheffel A Heidkamp R Xu YY Walton S Munos MK Advancing nutrition measurement: Developing quantitative measures of nutrition service quality for pregnant women and children in low- and middle-income country health systems. Matern Child Nutr. 2022;18:e13279. 10.1111/mcn.13279 34734469 PMC8710116

[24] World Health Organization. Survive and thrive: transforming care for every small and sick newborn. Geneva, Switzerland: World Health Organization; 2019. Available: https://www.who.int/publications/i/item/survive-and-thrive-transforming-care-for-every-small-and-sick-newborn. Accessed: 16 October 2025.

[25] Moxon SG Blencowe H Bailey P Bradley J Day LT Ram PK Categorising interventions to levels of inpatient care for small and sick newborns: Findings from a global survey. PLoS One. 2019;14:e0218748. 10.1371/journal.pone.0218748 31295262 PMC6623953

[26] Moxon SG Guenther T Gabrysch S Enweronu-Laryea C Ram PK Niermeyer S Service readiness for inpatient care of small and sick newborns: what do we need and what can we measure now? J Glob Health. 2018;8:010702. 10.7189/jogh.08.010702 30023050 PMC6038996

[27] Tunçalp Ӧ Were W MacLennan C Oladapo O Gülmezoglu A Bahl R Quality of care for pregnant women and newborns-the WHO vision. BJOG. 2015;122:1045–9. 10.1111/1471-0528.13451 25929823 PMC5029576

[28] Sheffel A Karp C Creanga AA Use of Service Provision Assessments and Service Availability and Readiness Assessments for monitoring quality of maternal and newborn health services in low-income and middle-income countries. BMJ Glob Health. 2018;3:e001011. 10.1136/bmjgh-2018-001011 30555726 PMC6267320

[29] Brizuela V Leslie HH Sharma J Langer A Tuncalp O Measuring quality of care for all women and newborns: how do we know if we are doing it right? A review of facility assessment tools. Lancet Glob Health. 2019;7:e624–32. 10.1016/S2214-109X(19)30033-6 30898495

[30] Nickerson JW Adams O Attaran A Hatcher-Roberts J Tugwell P Monitoring the ability to deliver care in low- and middle-income countries: a systematic review of health facility assessment tools. Health Policy Plan. 2015;30:675–86. 10.1093/heapol/czu043 24895350 PMC4421835

[31] The DHS Program. Introducing the Revised Service Provision Assessment (SPA). 13 May 2022. Available: https://blog.dhsprogram.com/introducing-the-revised-service-provision-assessment-spa/. Accessed: 23 June 2023.

[32] Mallick L Temsah G Wang W Comparing summary measures of quality of care for family planning in Haiti, Malawi, and Tanzania. PLoS One. 2019;14:e0217547. 10.1371/journal.pone.0217547 31173618 PMC6555515

[33] Sheffel A Zeger S Heidkamp R Munos MK Development of summary indices of antenatal care service quality in Haiti, Malawi and Tanzania. BMJ Open. 2019;9:e032558. 10.1136/bmjopen-2019-032558 31796487 PMC7003378

[34] Stierman EK Ahmed S Shiferaw S Zimmerman LA Creanga AA Measuring facility readiness to provide childbirth care: a comparison of indices using data from a health facility survey in Ethiopia. BMJ Glob Health. 2021;6:e006698. 10.1136/bmjgh-2021-006698 34610906 PMC8493923

[35] McCarthy KJ Blanc AK Warren C Bajracharya A Bellows B Validating women’s reports of antenatal and postnatal care received in Bangladesh, Cambodia and Kenya. BMJ Glob Health. 2020;5:e002133. 10.1136/bmjgh-2019-002133

[36] McCarthy KJ Blanc AK Warren CE Mdawida B Women’s recall of maternal and newborn interventions received in the postnatal period: a validity study in Kenya and Swaziland. J Glob Health. 2018;8:010605. 10.7189/jogh.08.010605 29904605 PMC5983915

[37] Carter ED Sheffel A Requejo J Kosek M Campbell H Eisele T Association between sick child facility readiness and quality of care at the individual and facility level in five low-and middle-income countries. BMC Health Serv Res. 2024;24:1400. 10.1186/s12913-024-11772-9 39538289 PMC11562504

[38] Do M Micah A Brondi L Campbell H Marchant T Eisele T Linking household and facility data for better coverage measures in reproductive, maternal, newborn, and child health care: systematic review. J Glob Health. 2016;6:020501. 10.7189/jogh.06.020501 27606060 PMC5012234

[39] Ameen S Siddique AB Peven K Rahman QS Day LT Shabani J Survey of women’s report for 33 maternal and newborn indicators: EN-BIRTH multi-country validation study. BMC Pregnancy Childbirth. 2021;21:238. 10.1186/s12884-020-03425-6 33765956 PMC7995710

[40] Kc A Peven K Ameen S Msemo G Basnet O Ruysen H Neonatal resuscitation: EN-BIRTH multi-country validation study. BMC Pregnancy Childbirth. 2021;21:235. 10.1186/s12884-020-03422-9 33765958 PMC7995695

[41] Rahman AE Hossain AT Zaman SB Salim N Kc A Day LT Antibiotic use for inpatient newborn care with suspected infection: EN-BIRTH multi-country validation study. BMC Pregnancy Childbirth. 2021;21:229. 10.1186/s12884-020-03424-7 33765948 PMC7995687

[42] Salim N Shabani J Peven K Rahman QS Kc A Shamba D Kangaroo mother care: EN-BIRTH multi-country validation study. BMC Pregnancy Childbirth. 2021;21:231. 10.1186/s12884-020-03423-8 33765950 PMC7995571

[43] Zaman SB Siddique AB Ruysen H Kc A Peven K Ameen S Chlorhexidine for facility-based umbilical cord care: EN-BIRTH multi-country validation study. BMC Pregnancy Childbirth. 2021;21:239. 10.1186/s12884-020-03338-4 33765947 PMC7995704

[44] Munos MK Maiga A Do M Sika GL Carter ED Mosso R Linking household survey and health facility data for effective coverage measures: a comparison of ecological and individual linking methods using the Multiple Indicator Cluster Survey in Côte d’Ivoire. J Glob Health. 2018;8:020803. 10.7189/jogh.08.020803 30410743 PMC6211616

[45] McCarthy KJ Blanc AK Warren CE Kimani J Mdawida B Ndwidga C Can surveys of women accurately track indicators of maternal and newborn care? A validity and reliability study in Kenya. J Glob Health. 2016;6:020502. 10.7189/jogh.06.020502 27606061 PMC5012235

[46] Blanc AK Diaz C McCarthy KJ Berdichevsky K Measuring progress in maternal and newborn health care in Mexico: validating indicators of health system contact and quality of care. BMC Pregnancy Childbirth. 2016;16:255. 10.1186/s12884-016-1047-0 27577266 PMC5006493

[47] Watt C Abuya T Warren CE Obare F Kanya L Bellows B Can reproductive health voucher programs improve quality of postnatal care? A quasi-experimental evaluation of Kenya’s safe motherhood voucher scheme. PLoS One. 2015;10:e0122828. 10.1371/journal.pone.0122828 25835713 PMC4383624

[48] Wehrmeister FC Barros AJD Hosseinpoor AR Boerma T Victora CG Measuring universal health coverage in reproductive, maternal, newborn and child health: An update of the composite coverage index. PLoS One. 2020;15:e0232350. 10.1371/journal.pone.0232350 32348356 PMC7190152

[49] Munos M, Sheffel A, Carter E, Perin J; The Improve Coverage Group. Methods for estimating maternal, newborn, and child health and nutrition effective coverage cascades from household and health facility surveys. medRxiv: 24319361v1 [preprint]. 2024. Available: https://www.medrxiv.org/content/10.1101/2024.12.20.24319361v1. Accessed: 16 October 2025 10.1101/2024.12.20.24319361

[50] Maternal and Child Survival Program. What Data on Maternal and Newborn Health Do National Health Management Information Systems Include? A review of data elements for 24 low and lower middle-income countries. 2025. [not available online].

[51] Maternal and Child Survival Program. What Data on Family Planning Are Included in National Health Management Information Systems? 2025. Available: https://mcsprogram.org/resource/what-data-on-family-planning-are-included-in-national-health-management-information-systems/. Accessed: 19 January 2025.

[52] United States Agency for International Development, Maternal and Child Survival Program. What Child Health & Nutrition Data Do 24 Countries' HMIS Include? Washington, D.C., USA: United States Agency for International Development; 2019. Available: https://www.childhealthtaskforce.org/resources/report/2019/what-child-health-nutrition-data-do-24-countries-hmis-include-mcsp-2019. Accessed: 19 January 2025.

